# Association of inflammatory gene polymorphisms with mechanical heart valve reoperation

**DOI:** 10.1186/s40064-016-2566-x

**Published:** 2016-06-30

**Authors:** Kyung Eun Lee, Joo Hee Kim, Jee Eun Chung, Gwan Yung Lee, Yoon Jeong Cho, Byung Chul Chang, Hye Sun Gwak

**Affiliations:** College of Pharmacy, Chungbuk National University, Cheongju, 28644 Korea; College of Pharmacy, Ajou University, Suwon, 16499 Korea; Division of Life and Pharmaceutical Sciences, College of Pharmacy, Ewha Womans University, 11-1 Daehyun-Dong Seodaemun-Gu, Seoul, 03760 Korea; Department of Thoracic and Cardiovascular Surgery, Yonsei University Medical Center, 50-1 Yonsei-Ro Seodaemun-Gu, Seoul, 03722 Korea

**Keywords:** Inflammatory gene polymorphisms, Mechanical heart valve, Reoperation, C-reactive protein

## Abstract

**Background:**

Various complications lead to reoperation in patients who undergo prosthetic valve replacement where inflammatory process could be involved. The goals of this study were to identify risk factors that correlate with reoperation in patients with prosthetic heart valves and to investigate the relationship between reoperation and inflammatory gene polymorphisms.

**Results:**

The study included 228 patients from the EwhA–Severance Treatment Group of Warfarin. Single nucleotide polymorphisms of c-reactive protein (*CRP*), interferon-gamma, interleukin 1 beta, interleukin 6, interleukin 10, transforming growth factor beta 1, and tumor necrosis factor genes were genotyped by means of SNaPshot and TaqMan assays. Thirty-nine patients (17.1 %) underwent more than one heart valve operation. A threefold increased risk for heart valve reoperation was evident in homozygous variant-type (TT) carriers as compared with ancestral allele carriers of *CRP* rs1205. Logistic regression analysis revealed that *CRP* rs1205 (OR 2.68, 95 % CI 1.22–5.90, p = 0.014), valve position (mitral valve OR 2.80, 95 % CI 1.01–7.80, p = 0.048; tricuspid valve OR 9.24, 95 % CI 2.46–34.70, p = 0.001; reference: aortic valve) and time after first operation (OR 1.13, 95 % CI 1.06–1.20, p < 0.001) affected the risk of reoperation.

**Conclusions:**

Inflammatory gene polymorphisms could be a possible marker of risk for reoperation in patients with prosthetic heart valve surgery.

## Background

Currently, approximately 280,000 prosthetic heart valves are implanted worldwide every year. Of these, about 50 % are mechanical valves (Pibarot and Dumesnil 2009). However, prosthetic heart valves may require one or more reoperations. About 10 % of the patients with an aortic or mitral mechanical valve usually undergo reoperation 10 years after the initial surgical procedure (Frank et al. 1995). Reoperations are more complicated than the initial valve operations, and are associated with higher mortality rates with mechanical valves than with tissue valves (Jones et al. 2001).

Various complications lead to reoperation in patients who undergo prosthetic valve replacement, the most frequent being obstructive valve thrombosis and pannus development (Rizzoli et al. 1999). Valve thrombosis occurs when a thrombus is attached to or is near a prosthetic heart valve, obstructing blood flow or causing valve regurgitation. Other reasons for reoperation include failure of previous valve repair, paravalvular leakage, infective or non-infective endocarditis, and prosthetic valve dysfunction due to fibrous tissue ingrowth and calcification (Piehler et al. 1995).

The infiltration of activated macrophages and T-cells, as well as cytokine release, has been described in human stenotic aortic valves. Previous studies have shown that cardiac valve calcification and ossification also involve an inflammatory process. This process includes the release of cytokines, chemokines, growth factors, and hydrolytic enzymes that contribute to angiogenesis, atherosclerotic plaque growth, and ossification of the valve (Helske et al. 2007). Although inflammation is assumed to be a factor in the occurrence of adverse complications after heart valve replacement (Sinning et al. 2012), studies to address the role of inflammatory gene polymorphisms related to valve surgery have been scarce. Therefore, this study was designed to determine the risk factors associated with reoperation in patients with prosthetic heart valves, with an emphasis on cytokine genetic polymorphisms, considering their role in inflammation-related prosthetic valve dysfunction.

## Methods

### Study population

The study population consisted of 228 patients from the EwhA–Severance Treatment (EAST) Group of Warfarin who underwent mechanical heart valve replacement between January 1982 and December 2009 at Severance Cardiovascular Hospital of Yonsei University College of Medicine in Seoul, Korea. EAST cohort is comprised of patients who had warfarin therapy after a mechanical heart valve replacement, and who did not have any evidence of hepatic or renal impairment. Patients were followed up continuously at the Severance Cardiovascular Hospital of Yonsei University Medical Center outpatient clinic. We reviewed the patients’ paper charts and electronic medical records from June 1983 through May 2013 and collected data on age, age at the time of operation, gender, time after the first heart valve surgery, position of the valve prosthesis, INR, and comorbidities. Blood samples used for genotyping were collected from the 228 patients during regular outpatient clinic visits. The study protocol was approved by the institutional review board and Ethics Committee of Severance Hospital (IRB No. 2009-4-0283), and informed consent was obtained from all patients prior to their participation in the study.

### Genotyping

Genomic DNA of study patients was prepared from ethylenediaminetetraacetic acid blood samples using a QIAamp DNA Blood Mini Kit (Qiagen, Hilden, Germany) according to standard procedures recommended by the manufacturer. We selected single nucleotide polymorphisms of C-reactive protein (*CRP*), interferon-gamma (*IFNG*), interleukin-1beta (*IL1B*), interleukin-6 (*IL6*), interleukin-10 (*IL10*), transforming growth factor beta-1 (*TGFB1*), and tumor necrosis factor (*TNFA*) based on previously published data regarding genetic polymorphisms in inflammation-related diseases, including coronary artery disease and myocardial infarction (Table 1; Cruz et al. 2013; Wang et al. 2015; Garg et al. 2013; Iacoviello et al. 2005). Genotyping was conducted using SNaPshot or TaqMan assays.

**Table 1:** SNP information

Gene rs number	Global minor allele frequency	Functional consequence	Direction	Primer sequence
*IL1B* rs16944	G = 0.4906	Upstream variant 2 KB	F^a^	CCAGCCAAGAAAGGTCAAT
			R^b^	GAAGAGGTTTGGTATCTGCCA
			G^c^	CAATTGACAGAGAGCTCC
*IL10* rs1800871	A = 0.4347	Upstream variant 2 KB	F	GAAACCAAATTCTCAGTTGGC
			R	ATGACCCCTACCGTCTCTATTT
			G	TGGTGTACCCTTGTACAGGTGATGTAA
*IL10* rs1800896	C = 0.2722	Intron variant	F	ACACACACACAAATCCAAG
			R	ATAGGAGGTCCCTTACTTTCCTC
			G	TCCTCTTACCTATCCCTACTTCCCC
*IFNG* rs2430561	A = 0.2802	Intron variant	F	ATATTCAGACATTCACAATTGATT
			R	TATTATACGAGCTTTAAAAGATAGTTCC
			G	TTTATXCTTACAACACAAAATCAAATC
*TNFA* rs1800629	A = 0.0903	Upstream variant 2 KB	F	AGAAGGAAACAGACCACAGAC
			R	GGGAAAGAATCATTCAACCA
			G	TAGGTTTTGAGGGGCATG
*TGFB1* rs1800470	G = 0.4547	Missense	F	GCCCATCTAGGTTATTTCC
			R	TGCCAGTCACTTCCTACC
			G	AGCAGCGGTAGCAGCAGC
*CRP* rs1205	T = 0.3383	UTR variant 3 prime	F	ATCTTYTTGCTGCTGGATTTC
			R	TTGTTTGTCAATCCCTTGG
			G	CTTGTTTGCCACATGGWGAGAGACT

^a^Forward primer

^b^Reverse primer

^c^Genotyping primer

### Statistical analyses

Univariate and multivariate analyses were performed to determine the association of these genotypes with the recurrence of heart valve operations. The Chi square test was used to compare categorical variables; if the expected frequencies were less than 5 in any one cell, we used Fisher’s exact test to obtain a p value. For continuous variables, an independent sample *t* test was performed, and their approximation to normality was judged by means of the Shapiro–Wilk W test. Depending on the distribution, either the *t* test or the Mann–Whitney test was used for analysis of the variables. A logistic regression model was used to investigate the factors that independently affected the number of operations. All analyses were performed with the SPSS Statistics version 20 software (IBM, New York City, NY, USA). Differences with a p value of less than 0.05 were considered statistically significant.

## Results

A total of 228 patients who had undergone heart valve replacements were followed-up from June 1983 to May 2013. Of these patients, 39 (17.1 %) underwent more than one heart valve operation. The reasons for reoperations were failure of the previous repair (n = 14), valve dysfunction (n = 17), periprosthetic leak (n = 5), valve thrombosis (n = 1), endocarditis (n = 1), and unknown (n = 1). There was a significant difference between the patients with and those without reoperation in the average length of time after the first surgery (p < 0.001). More female than male patients had undergone reoperation (20.8 vs. 10.1 %, p = 0.042). Valve positions were a statistically significant predictor of reoperation, and tricuspid valve surgery posed the highest risk for reoperation. There were no significant differences between patients with and those without reoperation in terms of age, age at operation, percentage of time in the therapeutic range on warfarin, and comorbidities. Table 2 shows the demographic characteristics with regard to the number of heart valve operations.

**Table 2:** Demographic characteristics and number of heart valve operations

Characteristics	Patient number or mean ± SD		p value
	Number of heart valve operations		
	1	≥2	
Operation	189	39	
Time after first operation (years)	16 ± 7	23 ± 9	0.002
Age	58 ± 10	57 ± 10	0.333
Age at first operation	43 ± 11	45 ± 11	0.476
Gender			
Male	71	8	0.042
Female	118	31	
Body mass index (kg/m^2^)	22.84 ± 2.77	21.70 ± 2.99	0.023
Time in therapeutic range on warfarin			
<60 %	132	27	0.866
≥60 %	55	12	
Valve position			
Aortic	52	5	0.001
Mitral	90	23	
Double^a^	38	3	
Tricuspid^b^	9	8	
Comorbidity			
Hypertension			
Absent	139	30	0.661
Present	50	9	
Diabetes mellitus			
Absent	174	34	0.326
Present	15	5	
Congestive heart failure			
Absent	146	34	0.166
Present	43	5	
Atrial fibrillation			
Absent	85	13	0.181
Present	104	26	
Myocardial infarction			
Absent	84	17	1.000
Present	105	22	

^a^Aortic plus mitral valve

^b^Any valve replacements including tricuspid valve

We evaluated the effects on reoperation of eight genetic variants of seven inflammatory mediator genes in 228 study patients. These genotypes included *IL1B* rs16944, *IL6* rs1800796, *IL10* rs1800871, *IL10* rs1800896, *IFNG* rs2430561, *TNFA* rs1800629, *TGFB1* rs1800470, and *CRP* rs1205. The observed genotype frequencies were consistent with the Hardy–Weinberg equilibrium for all SNPs. A statistically significant association between genotypes and reoperation was found for *IL10* rs1800896 and *CRP* rs1205. We observed a 3.3-fold increased risk for reoperation of the heart valve in homozygous variant-type (*TT*) carriers as compared with ancestral carriers of *CRP* rs1205. Two patients who carried *CC* allele of IL10 rs1800896 underwent reoperation, but there were no *CC* allele carriers in the single operation group. In contrast, 20.8 % of patients who carried the T allele underwent reoperation. Table 3 shows the association between reoperation and the grouped genotypes.

**Table 3:** Association between reoperation and grouped genotypes of inflammatory mediators

Gene polymorphisms (minor allele frequency)	Patient number		p value	OR (95 % CI)
	Number of heart valve operations			
	1	≥2		
*IL1B* rs16944 (0.43)				
AA, AG	126	27	0.756	1
GG	63	12		0.889 (0.422–1.871)
*IL6* rs1800796 (0.27)				
GG	15	2	0.744	1
GC, CC	172	37		1.613 (0.534–7.359)
*IL10* rs1800871 (0.29)				
AA, AG	170	37	0.743	1
GG	15	2		0.613 (0.134–2.794)
*IL10* rs1800896 (0.07)^a^				
TT, TC	184	37	0.030	
CC	0	2		
*IFNG* rs2430561 (0.20)				
AA	154	31	0.574	1
AT, TT	31	8		1.282 (0.538–3.053)
*TNFA* rs1800629 (0.04)				
GG	173	37	0.548	1
GA, AA	16	2		0.584 (0.129–2.652)
*TGFB1* rs1800470 (0.47)				
GG, GA	145	29	0.584	1
AA	40	10		1.250 (0.562–2.780)
*CRP* rs1205 (0.40)				
CC, CT	129	15	0.001	1
TT	60	23		3.297 (1.606–6.766)

*OR* odds ratio, *CI* confidence interval, *IL1B* interleukin-1beta, *IL6* interleukin-6, *IL10* interleukin-10, *INFG* interferon-gamma, *TNFA*: tumor necrosis factor alpha, *TGFB1*: transforming growth factor-beta1, *CRP*: C-reactive protein

^a^Odds ratio was not calculated due to no patients in certain group

After adjustment for variables that were statistically significant on the univariate analyses (i.e., gender, time after first operation, body mass index, valve position, and *CRP* rs1205), the binary logistic regression analysis confirmed that time after first operation, valve position, and *CRP* rs1205 were significantly associated with reoperation (Table 4). Patients with tricuspid valve replacements were at 9.2-fold increased risk for reoperation, compared with those who underwent aortic valve replacements. Variant-type homozygote carriers of *CRP* rs1205 showed a statistically higher risk for reoperation than did the ancestral allele carriers (p = 0.014, adjusted OR 2.684, 95 % CI 1.222–5.895).

**Table 4:** Results of logistic regression analysis

Variables	Adjusted odds ratio (95 % CI)	p value
Gender		
Male	1	0.179
Female	1.970 (0.732–5.303)	
Time after first operation (year)	1.129 (1.063–1.199)	<0.001
Body mass index (kg/m^2^)	0.911 (0.765–1.086)	0.300
Valve position		
Aortic	1	
Mitral	2.804 (1.009–7.796)	0.048
Double	0.821 (0.185–3.648)	0.796
Tricuspid	9.244 (2.463–34.695)	0.001
*CRP* rs1205		
CC, CT	1	0.014
TT	2.684 (1.222–5.895)	

The odds ratio was adjusted for gender, time after first operation, body mass index, valve position, and *CRP* rs1205

Clinical characteristics of minor allele (TT) carriers of rs1205 were 55 females (66.3 %, p = 0.598), younger age groups (<65 years; 57.8 %, p = 0.466), mitral valve prosthesis (50.6 %), and with atrial fibrillation (61.4 %, p = 0.070). However, rs1205 variant was not significantly associated with clinical demographics except for reoperation.

Subgroup analyses were conducted to compare the reoperation rate in relation to the genotypes in patients who underwent mitral valve operations. The first heart valve operation in 113 patients involved the mitral valve, and 23 of these patients underwent reoperation. The presence of *TGFB1* rs1800470 and *CRP* rs1205 polymorphisms was statistically significant with respect to mitral valve reoperations (p = 0.043 and p = 0.031, respectively). In addition, patients with the variant-type allele (A) of *TGFB1* rs1800470 were at lower risk for reoperation compared with those who had ancestral homozygous alleles (G) (OR 0.377, 95 % CI 0.144–0.987; Table 5). Consistent with the whole-population analysis, the risk of reoperation was increased in the TT carriers of *CRP* rs1205 (OR 2.734, 95 % CI 1.073–6.969).

**Table 5:** Subgroup analysis of patients with mitral valve operation

Gene polymorphisms (minor allele frequency)	Patient number		p value	OR (95 % CI)
	Number of heart valve operations			
	1	≥2		
*IL1B* rs16944 (0.45)				
AA, AG	65	15	0.510	1
GG	25	8		1.387 (0.523–3.673)
*IL6* rs1800796 (0.26)				
GG	22	1	0.692	1
GC, CC	83	7		1.855 (0.217–15.886)
*IL10* rs1800871 (0.27)				
AA, AG	79	22	0.466	1
GG	9	1		0.399 (0.048–3.332)
*IL10* rs1800896 (0.06)^a^				
TT, TC	88	23	0.205	
CC	0	1		
*IFNG* rs2430561 (0.08)				
AA	76	20	1.000	1
AT, TT	13	3		0.877 (0.228–3.378)
*TNFA* rs1800629 (0.04)				
GG	80	23	0.471	1
GA, AA	9	1		0.409 (0.049–3.405)
*TGFB1* rs1800470 (0.46)				
GG, GA	20	10	0.043	1
AA	69	13		0.377 (0.144–0.987)
*CRP* rs1205 (0.40)				
CC, CT	61	10	0.031	1
TT	29	13		2.734 (1.073–6.969)

^a^Odds ratio was not calculated due to no patients in certain group

## Discussion

Advances in surgical techniques and progress in valve design and materials have improved the clinical outcomes of patients with heart valve diseases. However, the placement of prosthetic heart valves often requires one or more reoperations. Several studies have sought to identify factors related to the occurrence of prosthetic valve dysfunction. Although the main causes may vary with patient demographic characteristics, disease progression as well as prosthesis type, model, and implantation sites were found to be influential factors (Aydin and Yapici 2013; Weerasinghe et al. 1999; Chan et al. 2011). In this study, we focused on genetic risk factors to investigate the association of gene polymorphisms of inflammatory mediators with reoperation after prosthetic heart valve placement.

As has been noted by previous researchers, time after the initial operation also affected the risk of reoperation in our study patients (Rahimtoola 2010; Khan et al. 2001). In addition, based on the results of our univariate analysis, gender, body mass index, and valve position were found to correlate with the risk for reoperation.

Conflicting results have been reported regarding the effect of gender on the risk of valve surgery (Lytle et al. 1986; Cohn et al. 1993). However, it is known that management and outcomes of valvular surgery can differ between men and women owing to differences in pathophysiology and body size (Redberg and Schiller 2004).

Consistent with previous findings, valve position affected risk of reoperation, showing highest risk in tricuspid valve replacement possibly because of the relatively high incidence of thrombosis or pannus ingrowth (Jones et al. 2001).

Although body weight has been shown to be associated with inflammation in previous studies, we found the reverse trend in our study (Fogarty et al. 2008; Nicklas et al. 2004). Obesity was not found to be a risk factor for adverse outcomes after valve surgery in other studies; however, our study confirmed that body mass index was associated with reoperation when analyzed as a continuous variable (p = 0.023) but not as a categorical variable using a cutoff of 25 kg/m^2^ (p = 0.069) (Allama et al. 2013). An absolute comparison cannot be made between our results and previous studies because the average body mass index of our patients in both groups was within normal range (20.0–24.9 kg/m^2^) and was rather lower on average in our study group as a whole (mean = 22.7 ± 2.8 kg/m^2^).

Insufficient anticoagulation or fluctuations in INR values within the therapeutic range can cause obstructive valve dysfunction. Therefore, we evaluated the effects of time to therapeutic range on the need for reoperation. A TTR cutoff value of 60 % was used based on previous studies in which patients with a TTR below 55–60 % had higher mortality and a higher incidence of bleeding (White et al. 2007). However, our results did not indicate that TTR was a predictor of reoperation (p = 0.866).

One of the main observations in this study was that reoperation was associated with inflammatory gene polymorphisms. Among the inflammatory mediators, *CRP* rs1205 was found to be associated with reoperation based on our final logistic regression model. Although SNPs of the other inflammatory mediator genes we tested were not statistically significant, we cannot exclude other genes from among the risk factors for reoperation, because allele frequencies can vary among ethnic groups, thus contributing to differences in outcomes.

Following the implantation of foreign material, acute and chronic inflammation can occur due to mediators such as interleukins, histamines, and growth factors. Moreover, inflammatory responses may affect the durability of prosthetic valves because thrombosis and degradation often occur in response to the activation and aggregation of platelets and inflammatory cells (Alavi et al. 2013). Many ongoing studies have explored the association between *CRP* genetic polymorphisms and their contribution to disease risk. One study found that the minor allele of the rs1205 *CRP* polymorphism could serve as a potential marker in identifying subjects prone to severe and heavily calcified aortic stenosis (Wypasek et al. 2015). Another study found that rs1205 was associated with a decreased risk of cardiovascular mortality in Caucasians (Lange et al. 2006). On the other hand, a huge randomization meta-analysis conducted by Wensley and colleagues showed that CRP levels genetically determined by *CRP* polymorphisms including rs1205 are unrelated to risk for coronary heart disease (Wensley et al. 2011). These conflicting results are not surprising, because just as the distribution of *CRP* alleles differs among races, disease risk also differs among races.

At present, the *CRP* rs1205 polymorphism is related to prosthetic valve dysfunction in Koreans patients who have undergone mechanical heart valve replacement, which presumably involves inflammatory responses. At this point, it is not easy to explain the mechanism underlying the observed association between the rs1205 polymorphism and heart valve reoperation. Previous researches reported that CRP, a biomarker of inflammation, was associated with valve stenosis which involves calcification and activation of local and systemic inflammation. *CRP* rs1205 mutation was associated with elevated CRP levels in patients with valve stenosis, suggesting inflammatory response and which can further be related with reoperation. Studies on the mechanism governing the contribution of rs1205 mutation to the need for reoperation remains to be determined.

The possible functionality of 3′-UTR rs1205 polymorphism affecting the reoperation could involve increased inflammatory cytokines. 3′-UTR mutations can affect mRNA stability of genes harboring them. The 3′-UTR of *CRP* is quite long suggesting its special regulatory importance. A mechanism involving mRNA stability would be the effect on microRNA binding sites as 3′-UTR could be the main target leading to a change of post-transcriptional regulation of gene expression. Another possible option would be that rs1205 polymorphism influences CRP expression by its linkage disequilibrium with another functional genetic variant. The complex haplotypic relationships can functionally influence the effects of rs1205 mutation (Potaczek et al. 2012; Russell et al. 2004; Di Marco et al. 2001).

In the analysis of the subgroup of patients with mitral valve operation, *TGFB1* rs1800470 was found to be statistically significant (p = 0.043). One study suggested that the *TGFB1* T29C (rs1800470) gene polymorphism could be associated with the risk of restenosis after coronary stent placement (Fragoso et al. 2015). This allele was previously associated with the risk of silent myocardial ischemia and acute coronary syndrome (Cruz et al. 2013; Syrris et al. 1998). Functional studies investigating the underlying mechanism revealed that the presence of the T allele produces a binding site for SF2/ASF proteins, which belong to the family of serine/arginine-rich (SR) proteins that regulate alternative splicing, suggesting that the *TGFB1* T29C polymorphism could have a functional effect (Syrris et al. 1998; Sureau et al. 2001).

There are several limitations of this study. This is a retrospective study in a single center using a single ethnic group. Detailed inflammatory clinical phenotypes such as CRP levels or pathological findings could not be gathered.

## Conclusions

In conclusion, this study is the first to demonstrate a relationship between inflammatory gene polymorphisms and heart valve reoperation. Genetic polymorphisms of *CRP* rs1205 and *TGFB1* rs1800470 were found to be factors influencing the need for reoperation, indicating the value of inflammatory gene variants as a possible marker for reoperation in patients who undergo prosthetic heart valve surgery. Given that this study was carried out at a single center and that the sample size was relatively small, our hypothesis requires further independent validation in multi-center studies with larger sample sizes of diverse ethnic groups.

## Abbreviations

CRP: c-reactive protein
IL1B: interleukin-1beta
IL6: interleukin-6
IL10: interleukin-10
INFG: interferon-gamma
INR: international normalized ratio
OR: odds ratio
SNP: single nucleotide polymorphism
TGFB1: transforming growth factor-beta-1
TNFA: tumor necrosis factor-alpha
TTR: time to therapeutic range
